# Proteome dataset of liver from dairy cows experiencing negative or positive energy balance at early lactation

**DOI:** 10.1016/j.dib.2021.107517

**Published:** 2021-10-27

**Authors:** Turner H. Swartz, Uzi Moallem, Hadar Kamer, Gitit Kra, Yishai Levin, Laman K. Mamedova, Barry J. Bradford, Maya Zachut

**Affiliations:** a Department of Animal Sciences, Michigan State University, East Lansing, MI 48824, USA; b Department of Ruminant Sciences, Institute of Animal Science, ARO Volcani Center, 68 HaMaccabim Road, P.O.B 15159, Rishon Lezion 7505101, Israel; c Department of Animal Science, the Robert H. Smith Faculty of Agriculture, Food and Environment, the Hebrew University of Jerusalem, Rehovot 76100, Israel; d The Nancy and Stephen Grand Israel National Center for Personalized Medicine, Weizmann Institute of Science, Rehovot 7610001, Israel

**Keywords:** Postpartum, Proteomics, Metabolism, Hepatic, Bovine

## Abstract

This article contains raw and processed data related to research published by Swartz et al. [1]. We present proteomics data from liver of postpartum dairy cows that were obtained by liquid chromatography-mass spectrometry following protein extraction. Differential abundance between liver of cows experiencing either negative energy balance (NEB, *n* = 6) or positive energy balance (PEB, *n* = 4) at 17 ± 3 days in lactation was quantified using MS1 intensity based label-free. There is a paucity of studies examining the associations of NEB with the liver proteome in early lactation dairy cows. Therefore, our objective was to examine the differences in the liver proteome in periparturient dairy cows experiencing naturally occurring NEB compared to cows in PEB. In this study, multiparous Holstein dairy cows were milked either 2 or 3 times daily for the first 30 days in milk (DIM) to alter energy balance, and were classified retrospectively as NEB (*n* = 18) or PEB (*n* = 22). We collected liver biopsies from 10 cows (*n* = 5 from each milking frequency), that were retrospectively classified according to their energy balance (NEB, *n* = 6; PEB, *n* = 4). The liver proteome was characterized using label-free quantitative shotgun proteomics. This novel dataset contains 2,741 proteins were identified, and 68 of those were differentially abundant between NEB and PEB (*P ≤* 0.05 and FC± 1.5); these findings are discussed in our recent research article [1]. The present dataset of liver proteome can be used as either biological markers for disease or therapeutic targets to improve metabolic adaptations to lactation in postpartum dairy cattle. Data are available via ProteomeXchange with identifier PXD028124.

## Specifications Table

**Table:** 

Subject	*Animal Science*
Specific subject area	*Dataset of liver proteome from postpartum cows that were classified as those in negative or positive energy balance*
Type of data	Table
How the data were acquired	Liquid Chromatography-Mass spectrometry: nanoAcquity+Q Exactive Plus
Data format	Raw Analyzed
Parameters for data collection	Multiparous Holstein dairy cows were milked either 2 or 3 times daily for the first 30 days in milk (DIM) to alter EB, and were classified retrospectively as NEB (*n* = 18) or PEB (*n* = 22). Liver biopsies were collected from 10 postpartum dairy cows at 17±3 DIM (5 from each milking frequency), that were retrospectively classified according to their energy balance, and subjected to proteomic analysis.
Description of data collection	Liver tissue was collected in vivo from 10 cows (*n* = 5 from each milking frequency) at 17±3 DIM, that were retrospectively classified according to their energy balance (NEB, *n* = 6; PEB, *n* = 4). Liver tissues were analyzed by Liquid Chromatography-Mass spectrometry following protein extraction. Differential abundance was quantified using MS1 intensity based label-free.
Data source location	• *Institution: ARO Volcani Center* • *City/Town/Region: Rishon Lezion* • *Country: Israel* • *Latitude and longitude for collected samples: latitude: 31.989347, longitude: 34.820444*
Data accessibility	With the article. Data are available via ProteomeXchange with identifier PXD028124.
Related research article	*T. H. Swartz, U. Moallem, H. Kamer, G. Kra, Y. Levin, L. K. Mamedova, B. J. Bradford, M. Zachut, Characterization of the liver proteome in dairy cows experiencing negative energy balance at early lactation. J. of Proteomics 246 (2021) 104308.*

## Value of the Data

• This work provides the first documentation of proteome dataset from liver of postpartum dairy cows that were in negative or positive energy balance, quantifying 2,741 proteins.
• The proteome dataset from bovine liver can be used as either biological markers for disease or as therapeutic targets to improve metabolic adaptations to lactation in postpartum dairy cattle.
• Differential abundance of 68 proteins was found between cows experiencing a negative vs. a positive energy balance postpartum. Further research can be done to explore the characteristics and functioning of these proteins in the inflammatory and metabolic processes in liver of dairy cows.

## 1 Data Description

This data describes the proteome of liver from postpartum dairy cows that were either in positive (PEB; 2.2 ± 0.6 Mcal/d) or negative energy balance (NEB; LSM ± SE, −6.3 ± 0.5 Mcal/d) postpartum (*P* < 0.0001) at the time of biopsy. **Supplementary Table 1** contains the dataset of 2,741 identified and quantified proteins obtained by proteomic analysis, as well as statistical analysis of differentially abundant proteins between liver of NEB and PEB cows. The full list of peptides is shown in **Supplementary Table 2**.

## 2 Experimental Design, Materials and Methods

### 2.1 Animals and procedures

The experimental protocol and procedures were approved by the Volcani Center Animal Care Committee (IL 637/16). The experiment was conducted at the experimental dairy farm of the Volcani Center, Rishon LeZion, Israel. The data represented within this paper are part of a larger study published elsewhere [2]. Full details on animal management and handling are provided in the companion paper [1]. In short, forty-two multiparous Holstein cows were divided into 2 subgroups: 21 cows were milked 3 times a day, and 21 cows were milked twice a day until 30 DIM; then, from 30 DIM, all cows were milked thrice daily. We expected that the different milking frequency would affect milk yield and feed intake and thus affect their energy balance, creating cows with varied energy balance. Postpartum, the cows were fed a common Israeli milking cow's ration. The energy balance was calculated according to NRC (2001) for the first 21 d of lactation, and cows were divided post-factum into 2 groups. During the first 21 DIM, the median of the average energy balance was −2.8 Mcal/d. Cows were classified as being in negative energy balance (NEB, n = 18) if the average energy balance during the first 21 DIM was less than −2.8 Mcal/d, and as being in positive energy balance (PEB, n = 22) if the mean energy balance during the first 21 DIM was greater than or equal to −2.8 Mcal/d. Two cows were excluded from the analysis due to extreme negative energy balance.

In a subset of ten cows, liver biopsies were performed (*n* = 5 from each milking frequency), that were retrospectively classified according to their energy balance (NEB, *n* = 6; PEB, *n* = 4), at 17 ± 3 days postpartum. To conduct the liver biopsy, the 11th intercostal space was shaved, sanitized, and then anesthetized with a 7-mL subcutaneous injection of 2% lidocaine HCl (Esracain 2%, 200 mg per 10 mL; Rafa Laboratories Ltd., Jerusalem, Israel). Then, an incision of ∼1 cm was made through the skin, and then the biopsy instrument (Bard Magnum; Bard Biopsy Systems, Tempe, AZ, USA) using a 14 G × 20 cm needle pierced the intercostal space. The procedure was guided by ultrasound (Aquila; Pie Medical Imaging BV, Maastricht, the Netherlands). We collected 3 to 4 liver samples from each cow (∼25 mg each)immediately snap frozen them in liquid nitrogen and stored at −80 °C. Then, the incision site was stapled, and treated topically with iodine spray. Staples were removed from cows at 7 to 10 d later.

### 2.2 Sample preparation for proteomic analysis

Protein concentrations in liver samples were determined by the bicinchoninic acid assay. Then, samples were subjected to tryptic digestion using a modified filter-aided sample preparation protocol. Samples were lysed in 1 mL SDT lysis buffer (4% SDS, 100mM Tris pH 7.6, DTT 100 mM) for 6 min at 95 °C, and cell debris was removed by centrifugation (16,000 × g, 10 min). Following that, 50 ¼g of each sample was taken from the supernatant, mixed with 200 ¼L urea buffer I (8.0M urea in 0.1M Tris–HCl pH 8.0), loaded onto a 30-kDa molecular-weight-cutoff filter (vivacon 500, VN01H22, Sartorius, Göettingen, Germany) and then centrifuged for 30 min at 14,000 × g. This was followed by one wash with urea buffer I and then centrifuged 30 min at 14,000 × *g*. Iodoacetamide was added on the filter, incubated for 10 min and centrifuged 20 min at 14,000 × g. Then, samples were washed twice using 200 ¼L ammonium bicarbonate. Following this, we added trypsin (1 ¼g) in 40 ¼L ammonium bicarbonate, and then samples were incubated at 37 °C overnight. Digested proteins were centrifuged, acidified with trifluoroacetic acid, and desalted in a solid-phase extraction column (Oasis HLB, Waters, Milford, MS, USA). Samples were stored at −80 °C until further analysis.

### 2.3 Liquid chromatography

For all chromatographic steps, Ultra LC–MS-grade solvents were used (Bio-Lab, Jerusalem, Israel). Each sample was subjected to split-less nano ultra-performance liquid chromatography (UPLC; 10 K psi nanoAcquity, Waters). The mobile phases were: (A) H_2_O+0.1% (v/v) formic acid and (B) acetonitrile +0.1% formic acid. Samples were desalted online using a reverse-phase C18 trapping column (180-¼m internal diameter, 20 mm length, 5 ¼m particle size; Waters). The peptides were then separated using an HSS T3 nano-column (75 ¼m internal diameter, 250 mm length, 1.8 ¼m particle size; Waters) at 0.35 ¼L/min. Peptides were eluted from the column into the mass spectrometer using the following gradient: 4 to 20% solution B in 150 min, 20 to 90% B in 15 min, maintained at 90% B for 5 min, and then back to initial conditions.

### 2.4 MS

The nano-UPLC was coupled online through a nano-ESI emitter (10 ¼m tip; New Objective, Woburn, MA) to a quadrupole orbitrap mass spectrometer (Q Exactive Plus, Thermo Scientific, Watham, MA) using a FlexIon nanospray apparatus (Thermo Scientific). Data were acquired in data-dependent acquisition mode using a Top20 method. The quadrupole isolation window was set to 1.7 mass units, MS1 resolution was set to 70,000 (at 200 m/z) with an automatic gain control (AGC) target of 3e6, and maximum injection time was set to 20 ms. MS2 resolution was set to 17,500 with an AGC target of 1e6 and a maximum injection time of 60 ms, and normalized collision energy was set to 26. Singly charged ions were excluded and dynamic exclusion was set to 60 s.

### 2.5 Data processing and analysis

Raw data was processed with MaxQuant v1.6.6.0. The data was searched with the Andromeda search engine against the bovine sequences from UniprotKB, version 2015_07, including both reviewed and unreviewed sequences and appended with common lab protein contaminants for a total of 23,970 sequences. Enzyme specificity was set to trypsin and up to two missed cleavages were allowed. Fixed modification was set to carbamidomethylation of cysteines and variable modifications were set to oxidation of methionines, deamidation of N or Q. Peptide precursor ions were searched with a maximum mass deviation of 4.5 ppm and fragment ions with a maximum mass deviation of 20 ppm. Peptide and protein identifications were filtered at an FDR of 1% using the decoy database strategy. The minimal peptide length was 7 amino-acids. Then, peptide identifications were propagated across samples using the match-between-runs option checked. Searches were performed with the label-free quantification option selected. The quantitative comparisons were calculated using Perseus v1.6.2.3. A Student's *t*-Test, after logarithmic transformation, was used to identify significant differences across the biological replica. Fold changes were calculated based on the ratio of geometric means of the case versus control samples. We excluded one outlier sample from analysis based on PCA analysis.

The mass spectrometry proteomics data have been deposited to the ProteomeXchange Consortium via the PRIDE [3] partner repository with the dataset identifier PXD028124.

### 2.6 Statistical analysis

Proteomics data, after logarithmic transformation, were analyzed using the Student's *t*-test (Genedata) to examine the associations with energy balance. Fold change was calculated as the ratio of arithmetic means of the intensities of NEB versus PEB samples. Proteins were considered as differential at *P* ≤ 0.05 and fold change (FC) ± 1.5.

## Ethics Statements

The experiment complied with the ARRIVE guidelines and were carried out in accordance with the National Institutes of Health guide for the care and use of laboratory animals (NIH Publications No. 8023, revised 1978).

## CRediT authorship contribution statement

**Turner H. Swartz:** Data curation, Formal analysis, Visualization, Writing – original draft. **Uzi Moallem:** Conceptualization, Data curation, Formal analysis, Funding acquisition, Investigation, Methodology, Project administration, Resources, Supervision, Writing – review & editing. **Hadar Kamer:** Data curation, Methodology, Project administration. **Gitit Kra:** Data curation, Formal analysis, Methodology. **Yishai Levin:** Data curation, Formal analysis, Methodology, Validation. **Laman K. Mamedova:** Investigation, Writing – review & editing. **Barry J. Bradford:** Visualization, Writing – review & editing. **Maya Zachut:** Conceptualization, Data curation, Formal analysis, Funding acquisition, Investigation, Methodology, Project administration, Resources, Software, Supervision, Validation, Visualization, Writing – review & editing.

## Declaration of Competing Interest

The authors declare that they have no known competing financial interests or personal relationships that could have appeared to influence the work reported in this paper.

## Appendix Supplementary materials

Image, application 1

Image, application 2
